# The balance of rumen degradable protein and readily available carbohydrate in sheep rations on *in vitro* fermentability

**DOI:** 10.5455/javar.2023.j729

**Published:** 2023-12-31

**Authors:** Yunilas Yunilas, Mardiati Zain, Ujang Hidayat Tanuwiria, Jasmal Ahmari Syamsu

**Affiliations:** 1 Department of Animal Science, Faculty of Agriculture, Universitas Sumatera Utara, Medan, Indonesia; 2 Department of Animal Nutrition, Faculty of Animal Science, Universitas Andalas, Padang, Indonesia; 3 Department of Animal Nutrition and Feed Technology, Faculty of Animal Science, Universitas Padjadjaran, Bandung, Indonesia; 4 Department of Animal Nutrition and Feed Science, Faculty of Animal Science, Universitas Hasanuddin, Makassar, Indonesia

**Keywords:** Balance, *in vitro*, RAC, RDP, rumen, sheep

## Abstract

**Objective:**

Protein and carbohydrates are substances needed by ruminants, especially sheep. Providing protein and carbohydrates must pay attention to their degradation. In addition, balancing nutrients to meet the nutritional needs of rumen microbes is very important because the unbalanced availability of rumen degradable protein (RDP) and readily available carbohydrate (RAC) at one time can cause suboptimal microbial protein synthesis efficiency.

**Materials and Methods:**

Completely randomized design with a nonfactorial pattern of five treatments with three replications. Treatment includes RDP and RAC ratios, namely R1 = 2.30, R2 = 2.00, R3 = 1.70, R4 = 1.50, and R5 = 1.30.

**Results:**

The results showed that the balance of RDP and RAC in sheep ration formulation *in vitro* had a very significant effect (*p* < 0.01) on NH_3_, microbial protein synthesis, total gas, total microbes, and organic matter digestibility (OMD) but had no significant effect (*p* > 0.05) on rumen pH and dry matter digestibility (DMD).

**Conclusion:**

The optimal balance of RDP and RAC in the formulation of sheep rations *in vitro* was obtained at a ratio of 2.30 with NH_3_ (mM) 8.47, rumen pH 5.97, microbial protein synthesis (mg/100 ml) 123, gas (ml/g of material) 145, total microbes (cells/ml) 2.012 × 10^6^, (log CFU cells/ml) 6.3025, DMD 61.0%, and OMD 63.1%.

## Introduction

Ruminant livestock, one of which is sheep, requires protein as the basis for the formation of body tissue and carbohydrates as an energy source, which is digested intensively in the rumen with the help of rumen microorganisms [1,2]. In addition, sheep feed is mostly a source of energy and low in protein, which will have an impact on the yield of fermented products in the rumen.

It is feared that providing feed protein without paying attention to the level of degradation will cause a deficiency in meeting the protein needs of microbes and livestock. In addition, differences in degradation rates vary and depend on the synchronized and comparative level of rumen protein degradation [3,4]. If substance N is degraded more quickly than the energy source (carbohydrates), then the ammonia resulting from the degradation of compound N will be transferred to the liver and then recycled to the digestive tract (a small part), where most of it is lost with urine secretion. rumen degradable protein (RDP) is a protein fraction that undergoes degradation by microbes in the rumen. An average of 50% of the microbial population in the rumen has protease enzymes to degrade protein feed sources. This protein fraction feed will quickly experience deamination by rumen microbial protease enzymes, resulting in NH_3_ [5].

Carbohydrate degradation must match the rate of protein degradation [6]. If the energy consumed is available in sufficient quantities, the livestock’s response to protein utilization will be better. High protein degradation produces NH_3_ high, but it must be balanced with the availability of easily digestible energy to produce carbon sources. RDP and readily available carbohydrate (RAC)-based ration formulations are considered effective because these rations will meet not only the needs of livestock but also the needs of microorganisms contained in the rumen. In addition, the unbalanced availability of RDP and RAC at one time can cause low microbial protein synthesis efficiency because microbial protein synthesis is influenced by RDP and RAC, so it is very important to balance nutrients to meet the nutrient needs of rumen microbes and high digestibility indicates the provision of RDP and RAC is sufficient for rumen microbial growth [7]. Based on this, the author conducted research regarding the balance of RDP and RAC in sheep rations and their *in vitro* fermentability.

## Materials and Methods

The ingredients used consist of elephant grass, *Indigofera*, coconut cake, corn, bran, tofu dregs, MC Dougall liquid, pepsin HCl 0.20% liquid, rumen fresh liquid, HgCl solution_2_ saturation, and distilled water.

The equipment used consists of a grinder, analytical balance, centrifuge, electric furnace, water shaker bath 39°C–40°C, measuring cup, porcelain cup, flask, thermometer, pH meter, Erlenmeyer, electric oven 105°C, desiccator, CO_2_ gas, filter paper, and Whatman.

The method used in this research is an experimental method using a completely randomized design with a nonfactorial pattern of five treatments with three replications. Treatment includes RDP and RAC ratios, namely R1 = 2.30 (RDP 60.4: RAC 26.0), R2 = 2.00 (RDP 60.3: RAC 30.2), R3 = 1.70 (RDP 60.5: RAC 35.5), R4 = 1.50 (RDP 60.0: RAC 40.1), and R5 = 1.30 (RDP 60.0: RAC 46.0).

### Ration formulation based on RDP and RAC ratio

The ration formulation based on the RDP and RAC ratio which will be tested *in vitro* on sheep, is shown in Tables 1–3.

### Preparation of feed ingredients

Preparation of feed ingredients starts with the drying process of elephant grass, *Indigofera*, and tofu dregs using an oven at 60°C for 24 h, as well as providing coconut meal, ground corn, fine bran, and ultra minerals. Then, the feed ingredients are floured using a grinder, then sifted using a 0.30 mm sieve. After all the feed ingredients are available, the process of mixing the feed ingredients until they are homogeneous is carried out, and the feed ingredients can be tested.

### In vitro testing

The complete feed sample preparation that has been ground is weighed at 1 gm and then put into the fermenter tube. Then, mix McDougall’s solution and rumen fluid in a ratio of 4:1 into a glass beaker and mix with CO_2_ gas. Then, the mixed solution is put into the fermenter tube, and the blank is made without adding any feed samples to the fermenter tube. Then, the fermenter tube is closed using an intermittent rubber stopper and inserted into the shaker water bath, which has been filled with water. The first incubation was carried out for 48 h with shaking at 80 rpm and a temperature of 39°C in a closed condition. After 48 h the sample was removed, the pH was measured, and several parts of the supernatant were separated for microbial calculations, followed by the addition of 1 ml of 5% HgCl_2_ and 2 ml 1 N Na_2_CO_3_ in each sample and blank. The sample was centrifuged at 2,500 rpm for 15 min, then the dissolved substance was discarded, and 40 ml of 0.20% pepsin solution was added to the fermenter tube. Samples were incubated again for 48 h. The sample was removed and centrifuged with the same settings, then the dissolved substance was discarded, and the residue was rinsed using distilled water and then centrifuged again. Samples were filtered using Whatman paper no. 41, then the sample was transferred to a cup and baked for 24 h at a temperature of 105°C. The samples that have been kilned are then burned in the kiln at a temperature of 600°C for 6–8 h until ash forms [8].

### Variables

The parameters measured are NH_3_, rumen pH, microbial protein synthesis, gas, total microbes, dry matter digestibility (DMD), and organic matter digestibility (OMD).

### Data analysis

The data were obtained, tabulated, and analyzed using variance. If real or very real results are obtained, then the Duncan multiple range test is carried out using SPSS software [9].

**Table 1.** Nutrient content of feed ingredients making up the test ration based on DM (%).

Feed	DM	OM	Ash	CP	CF	EE	NNFE	TDN	NDF	ADF
Elephant grass	93.0	87.0	13.0	13.0	31.0	2.61	40.5	57.2	67.2	40.1
*Indigofera*	90.0	91.4	8.59	30.9	17.4	2.39	41.0	67.6	23.2	21.0
Coconut cake	93.5	92.1	7.89	7.85	13.1	14.6	56.5	79.3	-	-
Corn	88.5	88.7	11.3	15.2	1.63	2.98	69.0	81.1	-	-
Bran	90.9	91.0	8.93	11.0	26.8	4.36	49.0	62.3	-	-
Tofu dregs	93.6	97.4	2.57	20.4	21.4	2.14	53.5	93.6	97.4	2.57

**Table 2.** Ration formulation based on RDP and RAC ratio for sheep.

Feed ingredients	R1	R2	R3	R4	R5
Elephant grass (%)	59	54	47	42	35
*Indigofera* (%)	14	12	10	8	7
Coconut meal (%)	10	11	14	12	15
Corn (%)	9	12	17	16	11
Bran (%)	3	6	9	13	16
Tofu dregs (%)	4	4	2	8	15
Mineral (%)	1	1	1	1	1
Total	100	100	100	100	100

**Table 3.** The nutritional content of sheep rations is based on the RDP and RAC ratio.

Nutritional content	R1	R2	R3	R4	R5
RDP	60.4	60.3	60.5	60.0	60.0
RAC	26.0	30.2	35.5	40.1	46.0
CP	15.3	14.9	14.3	14.4	14.4
TDN	63.0	63.9	65.5	65.6	66.0
CF	24.0	22.9	21.2	21.4	22.0
DM	88.0	88.0	88.1	88.7	90.0
OM	89.0	89.0	89.1	89.7	90.6
EE	3.82	4.01	4.45	4.25	4.62
NDF	43.0	39.1	33.9	30.1	49.9
ADF	26.5	24.1	20.9	18.5	25.1

**Table 4.** Composite fermentability of RDP and RAC of sheep rations *in vitro*.

Parameter	Treatment				
	R1	R2	R3	R4	R5
NH_3_ (mM)	8.47^A^	7.52^B^	8.35^A^	7.00^BC^	6.37^C^
pH rumen	5.97	5.60	5.50	5.73	5.37
Microbial protein synthesis (mg/100 ml)	123^A^	106^C^	110^B^	112^B^	101^D^
Gas (ml/gm of material)	145^D^	151^B^	147^C^	149^BC^	153^A^
Total microbes (cells/ml)	2.012 × 10^6^	1.909 × 10^6^	1.521 × 10^6^	1.549 × 10^6^	1.559 × 10^6^
Total microbes (log x, cells/ml)	6.3025^A^	6.2802^A^	6.1817^B^	6.1894^B^	6.1924^B^
DMD (%)	61.0	49.1	56.2	58.2	47.2
OMD (%)	63.1^A^	51.2^B^	60.2^A^	61.0^A^	53.0^B^

Note: different superscripts on the same line show very significant differences (*p* < 0.01).

## Results and Discussion

The RDP and RAC balance research produced NH_3_ ranging from 6.37 to 8.47 mM (Table 4). Many rumen microbes use NH_3_ to synthesize microbial proteins, which makes it an important indicator of the internal environment of the rumen [10]. NH_3_ is liberated and converted to ammonia [11]. Ration formulations with lower RDP and RAC ratios produce fluctuating NH_3_. This is thought to be because the RDP content in the ration fluctuates, where the R2, R4, and R5 treatments contain lower RDP than the R1 and R3 treatments, so that the levels of NH_3_ produced are also relatively low, or, in other words, the formation of NH_3_ is directly proportional to the availability of RDP in the ration. The NH3 levels produced are still below the levels required to support rumen microbial growth. This is in accordance with the statement of Xu et al. [12] that high-protein feed can increase NH_3_ concentrations in ruminant livestock. Reduce NH_3_ by encapsulating feed or providing a soluble energy source so that it can be used together with NH_3_ to synthesize microbial protein.

Based on diversity analysis, different RDP and RAC ratios in sheep ration formulations had a very significant effect (*p* < 0.01) on rumen NH_3_ levels. Duncan’s test showed that treatment R1 was not significantly different from R3 but was very different from R2, R4, and R5. This is thought to be because the provision of lower RDP, followed by the provision of higher RAC, does not support increased microbial protein synthesis. However, the NH_3_ produced is still within the normal range for rumen microbial growth. This is in accordance with the statement of Rosmalia et al. [13] that NH_3_ levels are 7.60–8.11 mM, which is within the normal range.

Table 4 shows the balance of RDP and RAC in sheep rations *in vitro* producing a pH ranging from 5.37–5.97. The resulting pH decreased from the pH before fermentation, namely 6.70–6.80. However, the pH is within the normal range. This is in accordance with the statement by Kitkas et al. [14] that rumen pH values range from 5.30 to 6.60, which is within the normal range. In addition, Asin et al. [15] added that the rumen pH value ranges from 5.50 to 7.50, which is within the normal range as well.

A pH that has decreased indicates that the hydrolysis process of organic compounds (RAC) such as carbohydrates has occurred into simple forms, namely organic acids volatile fatty acid (VFA). The accumulation of organic acids causes changes in rumen pH conditions. Apart from that, Saha et al. [16] state that lower feed intake, shorter rumination time, and lower saliva levels can all contribute to a decrease in rumen pH.

It appears that the lower the RDP and RAC balance ratio in the sheep’s ration, the lower the pH, or vice versa. This is related to microbial activity in hydrolyzing carbon sources. The decrease in pH occurs because microbial activity hydrolyzes carbohydrates and produces organic acids. Carbohydrates in the R5 treatment are relatively high compared to other treatments, thus allowing higher microbial activity to hydrolyze carbohydrates. This is reflected in the resulting pH being relatively lower than other treatments. According to the statement by Yunilas et al. [17], that is, the higher the microbial activity in hydrolyzing carbohydrates, the production of organic acids will increase so that the pH decreases. The accumulation of these organic acid products causes a decrease in the pH of the fermentation results.

Analysis of diversity showed that the ration formulation based on the RDP and RAC ratio of 2.30–1.30 had no significant effect (*p* > 0.05) on rumen pH. The pH achieved can support microbial protein synthesis but is still below normal microbial growth (pH 6.50). Suharti et al. [18] state that appropriate pH conditions indicate that the process of microbial growth and metabolism will not be disturbed so that the digestion process of the ration will be optimal.

The average microbial protein synthesis ranges from 101 to 123 mg/100 ml, or 10.1–12.3 mg/10 ml. The balance of RDP and RAC in sheep diets *in vitro* results in fluctuating microbial protein synthesis. Under constant RDP conditions, the higher the availability of RAC in the ration formulation, the lower the microbial protein synthesis, or vice versa, but high microbial protein synthesis was achieved in the treatment with an RDP and RAC ratio of 2.30, which was related to rumen N ammonia. This is in accordance with the statement of Lu et al. [4] that a significant increase in rumen ammonia N was associated with a significant increase in microbial protein synthesis results.

Analysis of diversity showed that the balance of RDP and RAC in sheep diets *in vitro* had a very significant influence (*p* < 0.01) on microbial protein synthesis. The average ratio of research results obtained is in the range of the research results of Zahera et al. [19], namely microbial protein synthesis values ranging from 12.0 to 15.1 mg/10 ml, but higher than research by Putri et al. [20]. This was due to the higher NH_3_ concentration in this study, meaning more nitrogen was available for microbial protein synthesis. The total concentration of VFA, which serves as an energy source and carbon skeleton, also influences microbial protein synthesis.

Formulation of sheep rations with RDP and RAC ratios ranging from 2.30 to 1.30 produces a total gas of 145–153 ml/gm feed (Table 4). It can be seen that the lower the RDP and RAC ratios of the treatment ration, the higher the total gas obtained, or vice versa. This shows an increasing trend along with a decrease in the RDP and RAC ratios.

Based on the diversity analysis, it was found that the ratio of RDP and RAC in the ration formulation had a very significant effect (*p* < 0.01) on the total gas produced. Duncan’s further tests showed that the R5 treatment was very significantly different from the other treatments. This is thought to be related to microbial activity in degrading carbon sources (RAC). High microbial activity occurs in treatments with high availability of carbon sources. High gas production indicates a high rate of degradation. According to the statement by Sun et al. [21], carbohydrate degradation produces products in the form of glucose, organic acids, and CO_2_.

The balanced RDP and RAC ratio of 2.30–1.30 in the *in vitro* ration resulted in total microbes fluctuating in the range of 1.521 × 10^6^–2.012 × 10^6^ cells/ml or 6.1817–6.3025 log CFU cells/ml (Table 4). Diversity analysis showed that different RDP and RAC ratios in the feed formulation had a very significant influence (*p* < 0.01) on the microbial (bacteria) population. The lower the RDP and RAC ratios, the lower the microbial (bacteria) population. This is related to the availability of easily degradable proteins and easily degradable carbon sources as providers of carbon frameworks for microbial (bacteria) growth.

Treatments R1 and R2 showed that the microbial (bacteria) population was significantly higher than the other treatments. This shows that RDP produces NH_3_ as an N contributor and requires RAC as a carbon source provider, which is not too high for optimal microbial protein synthesis.

The results of the research balance the availability of RDP and RAC at different ratios to DMD and OMD. DMD and OMD indicate the amount of feed that is degraded by rumen microbes and digested by postrumen enzymes [22]. Fermentability has a close relationship with the digestibility of feed ingredients or rations, especially the fermentation of carbohydrates, which are the largest nutrient component in ruminant feed. The easier it is for the ration to be fermented by microbes, the easier it will be to digest the ration. The fermentation process is able to reduce the crude fiber (CF) component [23].

The average dry matter (DM) digestibility fluctuates and shows an increasing trend (Table 4). This is in accordance with the statement of Sileshi et al. [24] that the high protein content of the diet shows that there are many nutrients, especially N-protein, that are available to be degraded and digested so that it can increase digestibility, improve microbial growth, and supply high levels of amino acids. Syamsi and Ifani [25] added that protein in the diet indicates the availability of N for rumen microbes, which can help microbial growth and the production of microbial synthesis when digesting nutrients. Therefore, increasing protein content in livestock diets can result in increased DMD and OMD.

It appears that the higher the RAC in the ration formulation, the lower the resulting ratio. A high RDP and RAC ratio has an influence on DMD. It is suspected that the optimum RDP and RAC ratio will stimulate microbial growth so that digestibility can be increased. The growth or increase in the population of microbes (bacteria) will indirectly increase the digestibility of feed ingredients. However, although there is a tendency for DMD to increase, it has not yet shown a significant effect. This can be seen from the results of the *in vitro* RDP and RAC synchronization diversity analysis in sheep diets, which did not have a significant effect (*p* > 0.05) on diet DMD.

The results of research on RDP and RAC ratios in ration formulations show that the average OMD fluctuates (Table 4). Based on diversity analysis, it was found that the balance of RDP and RAC had a very significant effect (*p* < 0.01) on OMD. Organic matter (OM) is part of the DM, and the OM components consist of CF, crude protein (CP), ether extract (EE), and non-nitrogen-free extract. Low DMD is followed by low OMD too.

It can be seen that treatments R1, R3, and R4 produce higher OMD than treatments R2 and R5. It is suspected that this is because microbial protein synthesis is relatively higher compared to treatments R2 and R5. Higher enzyme production is made possible by high microbial protein synthesis, which is positively correlated with the digestibility of organic feedstuffs. In addition, DMD is usually not always greater than OMD.

## Conclusion

The optimal balance of RDP and RAC in the formulation of sheep rations *in vitro* was obtained at a ratio of 2.30 with NH_3_ (mM) 8.47, rumen pH 5.97, microbial protein synthesis (mg/100 ml) 123, gas (ml/gm of material) 145, total microbes (cells/ml) 2.012 × 10^6^, total microbes (log x, cells/ml) 6.3025, DMD 61.0%, and OMD 63.1%.

## List of Abbreviations

CF, crude fiber; CP, crude protein; ^o^C, degree centigrade; DM, dry matter; DMD, dry matter digestibility; EE, ether extract; g, gram; h, hours; min, minutes; mg, milligram; mL, milliliter; mm, millimeter; OM, organic matter; OMD, organic matter digestibility; RAC, readily available carbohydrate; RDP, rumen degradable protein; rpm, revolutions per minute; VFA, volatile fatty acid.

## References

[1] Azizi-Shotorkhoft A, Sharifi A, Azarfar A, Kiani A. (2018). Effects of different carbohydrate sources on activity of rumen microbial enzymes and nitrogen retention in sheep fed diet containing recycled poultry bedding. J Appl Anim Res.

[2] Meehan DJ, Cabrita ARJ, Maia MRG, Fonseca AJM. (2021). Energy: protein ratio in ruminants: insights from the intragastric infusion technique. Animals.

[3] Acar MC. (2018). Determination of ruminal protein degradation of three forages using *in vitro* protein fractions and in situ protein degradability characteristics. J Dairy Vet Anim Res.

[4] Lu Z, Xu Z, Shen Z, Tian Y, Shen H. (2019). Dietary energy level promotes rumen microbial protein synthesis by improving the energy productivity of the ruminal microbiome. Front Microbiol.

[5] Hartinger T, Gresner N, Sudekum KH. (2018). Does intra-ruminal nitrogen recycling waste valuable resources? A review of major players and their manipulation. J Anim Sci Biotechnol.

[6] Okon P, Bachmann M, Wensch-Dorendorf M, Titze N, Rodehutscord M, Rupp C (2023). Feed clusters according to *in situ* and *in vitro* ruminal crude protein degradation. Animals.

[7] Anggraeny YN, Soetanto H, Kusmartono Hartutik (2015). Sinkronisasi suplai protein dan energi dalam rumen untuk meningkatkan efisiensi pakan berkualitas rendah. Wartazoa.

[8] Tilley JMA, Terry RA. (1963). A two-stage technique for the *in vitro* digestion of forage crops. J Br Grassl Soc.

[9] Adinurani PG. (2016). Design and analysis of agro trial data: manual and SPSS.

[10] Shen J, Zheng W, Xu Y, Yu Z. (2023). The inhibition of high ammonia to *in vitro* rumen fermentation is pH dependent. Front Vet Sci.

[11] Serra-Toro A, Vinardell S, Astals S, Madurga S, Llorens J, Mata-Alvarez J (2022). Ammonia recovery from acidogenic fermentation effluents using a gas-permeable membrance contactor. Bioresour Technol.

[12] Xu Y, Li Z, Moraes LE, Shen J, Yu Z, Zhu W. (2019). Effects of incremental urea supplementation on rumen fermentation, nutrient digestion, plasma metabolites, and growth performance in fattening lambs. Animals.

[13] Rosmalia A, Permana IG, Despal D. (2022). Synchronization of rumen degradable protein with non-fiber carbohydrate on microbial protein synthesis and dairy ration digestibility. Vet World.

[14] Kitkas GC, Valergakis GE, Kritsepi-Konstantinou M, Gelasakis AI, Katsoulos PD, Kalaitzakis E (2022). Association between ruminal pH and rumen fatty acids concentrations of holstein cows during the first half of laction. Ruminants.

[15] Asin J, Ramirez GA, Navarro MA, Nyaoke AC, Henderson EE, Mendonca FS (2021). Nutritional wasting disorders in sheep. Animals.

[16] Saha S, Gallo L, Bittante G, Schiavon S, Bergamaschi M, Gianesella M (2019). Rumination time and yield, composition, lactating holstein cows. Animal.

[17] Yunilas, Siregar AZ, Mirwhandhono E, Purba, Fati N, Malvin T. (2022). Potensi dan karakteristik larutan mikroorganisme lokal (MOL) berbasis limbah sayur sebagai bioaktivator dalam fermentasi. J Livest Anim Health.

[18] Suharti S, Aliyah DN, Suryahadi (2018). Karakteristik fermentasi rumen *in vitro* dengan penambahan sabun kalsium minyak nabati pada buffer yang berbeda. J Ilmu Nutr Teknol Pakan.

[19] Zahera R, Purwanti J, Evvyernie D. (2022). Populasi mikroba rumen, fermentabilitas, dan kecernaan suplementasi daun kelor dalam ransum sapi perah *in vitro*. J Ilmu Nutr Teknol Pakan.

[20] Putri EM, Zain M, Warly L, Hermon H. (2021). Effects of rumen degradable-to-undegradable protein ratio in ruminant diet on *in vitro* digestibility, rumen fermentation, and microbial protein synthesis. Vet World.

[21] Sun X, Cheng L, Jonker A, Munidasa S, Pacheco D. (2022). A review: plant carbohydrate types-the potential impact on ruminant methane emissions. Front Vet Sci.

[22] Putra LO, Suharti S, Sarwono KA, Sutikno S, Fitri A, Astuti WD (2023). The effects of heat-moisture treatment on resistant starch levels in cassava and on fermentation, methanogenesis, and microbial populations in ruminants. Vet World.

[23] Debi MR, Wichert BA, Liesegang A. (2019). Method development to reduce the fiber content of wheat bran and rice bran through anaerobic fermentation with rumen liquor for use in poultry feed. Asian-Australas J Anim Sci.

[24] Sileshi G, Mitiku E, Mengistu U, Adugna T, Fekede F. (2021). Effects of dietary energy and protein levels on nutrient intake, digestibility, and body weight change in Hararghe highland and Afar sheep breeds of Ethiopia. J Adv Vet Anim Res.

[25] Syamsi AN, Ifani M. (2023). Rumen fermentation profiles of protein-energy synchronization index-based ration: an *in vitro* study. JITV.

